# Avian Influenza A(H7N9) Virus Infection in Pregnant Woman, China, 2013

**DOI:** 10.3201/eid2002.131109

**Published:** 2014-02

**Authors:** Xian Qi, Lunbiao Cui, Ke Xu, Bin Wu, Fenyang Tang, Changjun Bao, Yefei Zhu, Ming-hao Zhou, Hua Wang

**Affiliations:** Jiangsu Provincial Center for Disease Control and Prevention, Nanjing, China

**Keywords:** influenza virus, avian influenza A(H7N9) virus, influenza, viruses, pregnant woman, China

**To the Editor:** In February 2013, human infection with reassortant avian influenza A(H7N9) virus occurred in eastern China. A total of 135 laboratory-confirmed cases and 44 deaths among case-patients have been reported as of August 11, 2013. Unlike infection with other H7 subtype viruses (e.g., H7N2, H7N3, and H7N7), which often cause mild-to-moderate-human disease (*1*), infection with H7N9 subtype virus caused severe pneumonia and acute respiratory distress syndrome in most laboratory-confirmed case-patients (*2**,**3*). Pregnant women are particularly susceptible to severe complications from influenza (seasonal and pandemic), and have an increased risk for maternal death (*4*).

On March 30, 2013, a 25-year-old pregnant woman came to the outpatient department of a hospital in Zhenjiang, Jiangsu Province, China. She had cough and fever (temperature 38.0°C), which had begun 2 days earlier. She also reported mild myalgia and mild sore throat. The patient had no any underlying medical conditions and was at 17 weeks gestation, as estimated by ultrasound. On April 5, she was admitted to the respiratory department of the hospital with a temperature of 39.9°C, a leukocyte count of 7.9 ×10^9^ cells/L, and a lymphocyte count of 0.7 × 10^9^ cells/L.

On April 6, she was transferred to the intensive care unit because of shortness of breath, respiratory failure, and loss of consciousness. She was given mechanical ventilation, broad-spectrum antimicrobial drugs, oseltamivir, gamma-globulin, antifibrotic therapy (glutathione), and nutritional support. Oseltamivir (150 mg/d, 2 times/d) had been administered during April 6–12. A chest radiograph showed extensive infiltrates of both lungs.

On April 21, she regained consciousness, and her condition stabilized over the next few days. On April 23, she was extubated, transferred to the common ward, and given nasal oxygen supplementation and antimicrobial and antifibrotic drug therapy. Her condition improved gradually, and on May 14 she was discharged in good health without fetal abnormality.

The fetus was monitored daily by using ultrasound to check the heart rate; fetal heart rate and activity were normal during hospitalization. The fetus continued to grow appropriately and was delivered by cesarean section at 35 weeks’ gestation on July 17 (length 48 cm, weight 3,300 g, and Apgars scores of 9 at 1 min and 10 at 5 min). The clinical timeline for the case-patient is shown in the Figure.

**Figure:**
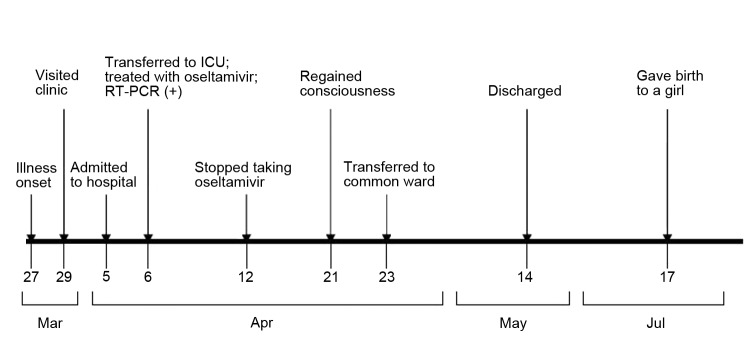
Clinical timeline for a pregnant woman infected with avian influenza A(H7N9) virus, China, 2013. ICU, intensive care unit; RT-PCR, reverse transcription PCR.

The patient and her husband lived in a house with her husband’s parents. No live poultry were present in the residential district, but the husband’s parents worked as pork butchers in a live animal market ≈500 m from the residential district. Several kinds of live poultry (e.g., chicken, duck, pigeon, and quail) were sold in the market. During the 2 weeks before illness onset, the patient did not have contact with persons known to be febrile. However, during that time, she visited the live animal market once. Eighteen potential close contacts of the patient were identified (15 health care workers and 3 household members). Respiratory symptoms did not develop in any of these contacts during a 7-day surveillance period.

Four methods were used for laboratory diagnosis: real-time reverse transcription PCR, virus isolation, full-genome sequencing, and modified hemagglutination inhibition assays. Clinical samples tested were 2 throat swab specimens obtained from the patient on April 6 and 7, 38 paired serum samples obtained from the patient and close contacts during the acute and convalescent phases of infection, and 6 environmental samples (2 avian feces samples and 4 poultry cage specimens obtained from the live animal market that the patient visited). Throat swab specimens from patient were positive for the hemagglutinin (HA) and neuraminidase genes of avian influenza A (H7N9) virus. Of 6 environmental samples, 5 were positive for (H7N9) virus HA genes. No (H7N9) virus HA antibodies were detected from paired serum samples from all 18 close contacts.

Two virus strains were isolated: 1 from a patient specimen (A/Zhenjiang/1/2013) and 1 from a chicken cage specimen (A/environment/Zhenjiang/4/2013) (GenBank accession nos. KF007057–KF007064 and KF007009–KF007016, respectively). Genome comparison showed that isolates had a nucleotide identity of 96.8%–99.8%, indicating an amino acid identity of 98_·_2%–99_·_6%. Phylogenetic analysis showed that 5 genes (HA, nucleoprotein, neuraminidase, matrix, and nonstructural protein) of the 2 isolates belonged to the same clade. However, the 3 polymerase genes (polymerase basic 1, polymerase basic 2, and polymerase acidic) clustered in a different clade. These results suggested that the 2 strains originated from an independent reassortment mechanism and that the H7N9 subtype viruses had undergone genetic reassortment to generate multiple novel genotypes in China.

According to epidemiologic and clinical data for infections with avian influenza A(H7N9) virus, most patients with severe illness, including severe pneumonia and acute respiratory distress syndrome, were elderly men with underlying medical conditions (*2**,**3*). Our findings suggest that pregnancy might be a risk factor for clinically severe influenza in young women infected with H7N9 subtype virus.
